# Changes in patterns of multimorbidity and associated with medical costs among Chinese middle-aged and older adults from 2013 to 2023: an analysis of repeated cross-sectional surveys in Xiangyang, China

**DOI:** 10.3389/fpubh.2024.1403196

**Published:** 2024-08-07

**Authors:** Changyu Ju, Hongjia Liu, Yongxiang Gong, Meng Guo, Yingying Ge, Yuheng Liu, Rui Luo, Meng Yang, Xiuying Li, Yangwenhao Liu, Xiangbin Li, Tiemei He, Xiaodong Liu, Chunrong Huang, Yihua Xu, Juming Liu

**Affiliations:** ^1^Party Office (United Front Work Department, Youth League Committee), Xiangyang Central Hospital, Affiliated Hospital of Hubei University of Arts and Science, Xiangyang, China; ^2^School of Accounting, Hunan University of Technology and Business, Changsha, China; ^3^Department of Medical Records and Statistics, Xiangyang Central Hospital, Affiliated Hospital of Hubei University of Arts and Science, Xiangyang, China; ^4^Division of Cardiac Surgery, Wuhan Asia Heart Hospital Affiliated with Wuhan University of Science and Technology, Wuhan, Hubei, China; ^5^Human Resources Department, Xiangyang Central Hospital, Affiliated Hospital of Hubei University of Arts and Science, Xiangyang, China; ^6^Information Center, Xiangyang Central Hospital, Affiliated Hospital of Hubei University of Arts and Science, Xiangyang, China; ^7^Neurology Department, Xiangyang Central Hospital, Affiliated Hospital of Hubei University of Arts and Science, Xiangyang, China; ^8^Department of Epidemiology and Health Statistics, School of Public Health, Tongji Medical College, Huazhong University of Science & Technology, Wuhan, China

**Keywords:** multimorbidity patterrn, middle-aged and older adults, medical cost, latent class analysis (LCA), China

## Abstract

**Background:**

Multimorbidity has become a major public health problem among Chinese middle-aged and older adults, and the most costly to the health care system. However, most previous population-based studies of multimorbidity have focused on a limited number of chronic diseases, and diagnosis was based on participants’ self-report, which may oversimplify the problem. At the same time, there were few reports on the relationship between multimorbidity patterns and health care costs. This study analyzed the multimorbidity patterns and changes among middle-aged and older people in China over the past decade, and their association with medical costs, based on representative hospital electronic medical record data.

**Methods:**

Two cross-sectional surveys based on representative hospital data were used to obtain adults aged 45 years and older in Xiangyang in 2013 (*n* = 20,218) and 2023 (*n* = 63,517). Latent Class Analysis was used to analyze changes in the patterns of multimorbidity, gray correlation analysis and ordered logistics model were used to assess the association of multimorbidity patterns with medical expenses. The diagnosis and classification of chronic diseases were based on the International Classification of Diseases, Tenth Revision codes (ICD-10).

**Results:**

The detection rate of chronic disease multimorbidity has increased (70.74 vs. 76.63%, *p* < 0.001), and multimorbidity patterns have increased from 6 to 9 (2013: Malignant tumors pattern, non-specific multimorbidity pattern, ischemic heart disease + hypertension pattern, cerebral infarction + hypertension pattern, kidney disease + hypertension pattern, lens disease + hypertension pattern; new in 2023: Nutritional metabolism disorders + hypertension pattern, chronic lower respiratory diseases + malignant tumors pattern, and gastrointestinal diseases pattern) in China. The medical cost of all multimorbidity patients have been reduced between 2013 and 2023 (RMB: 8216.74 vs. 7247.96, IQR: 5802.28–15,737 vs. 5014.63–15434.06). The top three specific multimorbidity patterns in both surveys were malignancy tumor pattern, ischemic heart disease + hypertension pattern, and cerebral infarction + hypertension pattern. Hypertension and type 2 diabetes are important components of multimorbidity patterns. Compared with patients with a single disease, only lens disorders + hypertension pattern were at risk of higher medical costs in 2013 (aOR:1.23, 95% CI: 1.03, 1.47), whereas all multimorbidity patterns were significantly associated with increased medical costs in 2023, except for lens disorders + hypertension (aOR:0.35, 95% CI: 0.32, 0.39). Moreover, the odds of higher medical costs were not consistent across multimorbidity patterns. Among them, ischemic heart disease + hypertension pattern [adjusted odds ratio (aOR):4.66, 95%CI: 4.31, 5.05] and cerebral infarction + hypertension pattern (aOR: 3.63, 95% CI: 3.35, 3.92) were the two patterns with the highest risk. Meanwhile, men (aOR:1.12, 95CI:1.09, 1.16), no spouse (aOR:1.09, 95CI: 1.03, 1.16) had a positive effect on medical costs, while patients with total self-pay (aOR: 0.45, 95CI: 0.29, 0.70), no surgery (aOR: 0.05, 95CI: 0.05, 0.05), rural residence (aOR: 0.92, 95CI: 0.89, 0.95), hospitalization days 1–5 (aOR: 0.04, 95CI: 0.04, 0.04), and hospitalization days 6–9 (aOR: 0.15, 95CI: 0.15, 0.16) had a negative impact on medical costs.

**Conclusion:**

Multimorbidity patterns among middle-aged and older adults in China have diversified over the past decade and are associated with rising health care costs in China. Smart, decisive and comprehensive policy and care interventions are needed to effectively manage NCDS and their risk factors and to reduce the economic burden of multimorbidity on patients and the country.

## Introduction

Increasing prevalence and burden of chronic non-communicable diseases (NCDS) in the context of a globally aging population (1, 2). NCDS were characterized by a long course of disease, complex etiology, and poor prognosis, seriously jeopardizing human health (3, 4). In 2008, the WHO defined chronic diseases as multimorbidity when a person with a chronic disease has two or more chronic diseases at the same time (5). Compared with patients with a single chronic disease, patients with multimorbidity will aggravate the difficulty of diagnosis and treatment (6), prolong the hospitalization time (7), lead to the use of joint medication (8, 9), increase medical consumption, aggravate the economic burden of the family and the society (10), and seriously affect the quality of life and life expectancy of middle-aged and older people (11).

Corresponding to the serious chronic disease epidemic is the increasing cost of healthcare. A long-term health and economic burden projection for chronic obstructive pulmonary disease in the United States showed that discounted direct medical costs attributable to COPD were estimated to be $80.90 billion from 2019 to 2038 (12). Global healthcare costs for treating people with diabetes reached $850 billion in 2017, accounting for 12.5% of global healthcare spending, with China ranking second in the world with $110 billion in diabetes healthcare costs (13). A global study of the economic burden of diabetes states that the economic burden of diabetes will reach 2.2% of global GDP in 2020 (14). WHO studies on the burden of NCDS showed that NCDS have an impact on the health-care expenditures of households, societies, and Governments, and ultimately on the macro economies of countries, with each 10 percent increase in chronic non-communicable diseases reducing the rate of growth of a country’s economy by 0.5 percent (15). Multimorbidity can increase the financial burden on patients. Studies in the United States have found that patients with two or three NCDS have medical costs that are 19% higher than those of patients with common chronic diseases. Furthermore, the cost of medical increases with the age of the patient (16). According to a study conducted by academics in England, healthcare costs increase as the number of co-morbidities increases. Patients with four or more chronic conditions have an average healthcare cost that is 5.2 times higher than that of patients without chronic conditions (17). A recent study in China based on 11 NCDS found that the prevalence of multimorbidity among people aged 50–54 years increased from 51 to 71% among people aged 75 years and older (18), and has implications for healthcare spending (19).

Given the different sociodemographic structures, disease patterns and health policy, the patterns of multimorbidity among middle-aged and older adults and medical costs in China may be different from those of other countries. Previous explorations of the relationship between multimorbidity and medical cost rarely investigated the relationship between diferent multimorbidity patterns and medical cost in China. Furthermore, the majority of data from previous multimorbidity surveys in China were usually obtained from community surveys, which may have resulted in recall and reporting deviations in the collected illness data. Additionally, many of the chronic illnesses that researchers focused on were predetermined during the study design, disregarding the potential impact of other chronic illnesses on multimorbidity patterns and medical costs. Moreover, there was limited data available on recent trends in multimorbidity patterns of chronic diseases in China, and their correlation with healthcare costs remained unexplored. Based on representative hospital electronic medical record data, this study reflects the disease status from the individual patient’s point of view. It includes all reported clinical diagnoses of chronic diseases in 2013 and 2023. The study analyzes the associations between changes in multimorbidity patterns and medical costs among middle-aged and older people in China over the past 10 years. These findings can be used as a reference for preventing and managing chronic diseases, reforming healthcare, and conducting related economic evaluations.

## Materials and methods

### Study design and participants

This is an analysis of repeated cross-sectional surveys based on hospital electronic medical record data in Xiangyang, China. Xiangyang is a vice-center city located in Hubei Province. It has a population of over 5 million and ranks first in gross domestic product among prefecture-level cities in central China. Xiangyang City governs three administrative districts (Xiangcheng, Fancheng, and Xiangzhou) and six counties (Zaoyang, Yicheng, Nanzhang, Baokang, Gucheng, and Laohekou). The study obtained hospitalization information from Xiangyang Central Hospital, the largest hospital in Xiangyang city, which provides over 2 million healthcare services annually. The study included a sufficient number of cases and cost analysis cases. A comparative analysis of repeated cross-sectional surveys was conducted by collecting information from the electronic medical records of all chronically ill inpatients in this hospital between 2013 and 2023. The exclusion criteria for this study were age less than 45 years, primary diagnosis not NCDS, incomplete information on the front page of the case and the content of the medical record, acute disease, all diagnoses without NCDS, and chronic disease detection rate less than 1%. In total, 20,218 and 63,517 valid eligible medical records were obtained between 2013 and 2023, respectively. This study did not involve clinical efficacy assessment, so no sample size calculations or non-inferiority tests were required. All data were collected from the hospital information system (Supplementary Figures S1, S2).

### Assessments of covariates, chronic conditions, and multimorbidity

Multimorbidity was defined as reporting co-existence of two or more chronic diseases/conditions within one person. Chronic conditions were determined by extracting the primary diagnosis and the first to ninth secondary diagnoses from the hospitalization information, and the diagnosis of chronic conditions was labeled according to the International Classification of Diseases, Tenth Revision codes (ICD-10). The study included age as a covariate, with age of 45–49, 50–59, 60–69, 70–79, and ≥ 80. Other covariates included sex, type of health insurance (UEBMI: urban employee basic medical insurance, URBMI: urban resident basic medical insurance, TSP: Total self-paying, CMI: Commercial medical insurance), spouse (yes or no), length of stay in hospital (1–5, 6–10, ≥10), surgery (yes or no), and type of residence (rural or urban).

Our primary outcome variables include the direct hospitalization costs, which are classified into comprehensive medical service fees (consultation fees, bed fees, and nursing fees), diagnostic costs (pathological diagnosis, laboratory diagnosis, imaging diagnosis, and clinical diagnosis, i.e., traditional examination and laboratory fees) with reference to the classification of hospitalization costs in the first page of China’s medical records, Treatment costs (non-surgical treatment costs, surgery costs, anesthesia costs, rehabilitation costs, and Chinese medicine treatment costs), medicine costs (western medicine costs, Chinese medicine costs, and blood and blood products category costs), consumables costs (disposable medical materials for examination, disposable medical materials for treatment, and disposable medical materials for surgery), and other costs (air-conditioning costs, escorting costs). Using the costs for 2023 as a benchmark, we normalized the costs for 2013 based on the Consumer Price Index. In addition, inpatient costs were classified into four classes (0–25, 25–50, 50–75%, and 75–100%) through quartiles, i.e., the cost ranges in 2013 were classified as less than RMB 5364.12 (Q1), RMB 5364.12–8303.73 (Q2), RMB 8303.73–14027.74 (Q3), and greater than 14,027.74 (Q4), and for 2023, the cost range is divided into RMB 4810.43 or less (Q1), RMB 4810.43 to RMB 7121.03 (Q2), RMB 7121.03 to RMB 13479.75 (Q3), and greater than RMB 13479.75 (Q4).

### Statistical analysis

Descriptive statistics were used to summarize the distribution of participants’ characteristics, with frequencies (percentage) for categorical variables and median (interquartile range, IQR) for continuous variables. The differences in observed prevalence of chronic diseases/conditions and multimorbidity between gender and residential regions were evaluated using Fisher exact tests or Chi-square.

Latent class analysis (LCA) is a robust statistical method employed to uncover latent categories demonstrating statistical correlations among categorical exogenous variables. Recent research has expanded its use to extensively explore patterns of multimorbidity within diverse populations (20). The primary objective of LCA is to elucidate the relationships between categorical variables by identifying the minimal number of potential categories necessary, while ensuring each category maintains local independence. The posterior probabilities of observed variables within each potential category are deduced by calculating the likelihood of each category and the conditional probabilities of the variables within those categories. Based on the magnitude of these posterior probabilities, the most probable category for a given combination of variables is ascertained. This method effectively creates a new categorical variable that captures the essence of the relationships among the observed values, thereby facilitating the clustering of the data into meaningful patterns. In the current study, gray correlation analysis (GRA) was performed to estimate the effect of hospitalization expenses on multimorbidity patterns. The calculation steps are as follows: ① Establish the original database, in this study, the total inpatient costs of multimorbidity patterns derived from the analysis of LCA and the costs of each classification were, respectively, selected as the evaluation indexes, and a gray correlation analysis database was established. ② Determine the reference series, selecting the total cost as the reference series and the six categorical costs as the comparison series. ③ The dimensionless processing of raw data can strengthen the comparability between indicators, and the commonly used methods include the initial value method and the mean value method. In this study, the mean value method was used to remove the dimension. ④ Calculate the absolute difference between each comparative series and the reference series, and find out the maximum and minimum values. ⑤ Calculate the correlation coefficient. ⑥ Find the degree of relatedness (R). Generally, 0 < R < 0.35 is considered as weak correlation, 0.35 ≤ R < 0.65 is considered as medium correlation, and 0.65 ≤ R < 1 is considered as strong correlation. Ordered multicategorical logistic regression were used to examine the relationship between hospitalization expenses and multimorbidity patterns, while accounting for other potential confounding variables as covariates (age, sex, health insurance type, spouse, hospitalization days, surgery, and residence type). All analyses were performed with R 4.3.1 (R Institute for Statistical Computing, Vienna, Austria). The packages “poLCA,” “quantreg,”“MASS,” and “forestploter” were applied.

### Sensitivity analyses

Sensitivity analyses were performed to assess the data robustness of the results. First, because previous studies have found a significant positive effect of age on health care expenditures (21), analyses were repeated excluding participants over age 80 (2013, *n* = 2,888, 2023, *n* = 4,510) to eliminate the effect of advanced age on the results. Second, because more than 90% of participants participated in either UEBMI or URBMI, analyses were repeated by only including individuals participating the above medicare modalities to reduce the misclassification bias. Finally, quantile regression models were used to assess the impact of comorbidity patterns on medical cost to test the robustness of the results between 2013 and 2023.

## Results

### Baseline characteristics of participants

Table 1 lists the participants’ baseline characteristics: in 2013, the study population had a median age of 61 (IQR: 53–69), with 50.88% being male. The total direct hospitalization cost was RMB 8303.74 (IQR: 5364.41–14,026), and the length of hospital days was 10 (IQR: 7–15). Of the population, 5.38% were aged ≥80, 13.86% lived in rural areas, 50.89% were aged ≥10 days, 5.25% were total self-paying, and 9.64% underwent surgery. In 2023, the study population had a median age of 64 (IQR: 56–71), with a total direct hospitalization cost of 7121.16RMB (IQR: 4810.56–13479.90) and the length of hospital days was 6 (IQR: 4–10). Of the population, 52.33% were male, 7.10% were aged ≥80 years, 55.32% lived in rural areas, 86.1% had spouses, 2.28% were fully self-pay, and 19.27% underwent surgery.

**Table 1:** Baseline characteristics of the participants.

Characteristics	2013 (*N* = 20,218)		2023 (*N* = 63,517)	
	*N*	Percentage (%)	*N*	Percentage (%)
Age (years) at admission				
45~	2,715	13.43	4,552	7.17
50~	6,218	30.75	17,792	28.01
60~	6,527	32.28	21,033	33.11
70~	3,670	18.15	15,629	24.61
≥80	1,088	5.38	4,510	7.10
Sex				
Men	10,287	50.88	33,236	52.33
Women	9,931	49.12	30,280	47.67
Health insurance type at admission				
UEBMI	14,294	70.70	27,724	43.65
URBMI	4,756	23.52	34,263	53.94
TSP	1,061	5.25	1,449	2.28
CMI	107	0.53	80	0.13
Spouse				
No	506	2.50	3,874	6.10
Yes	19,712	97.50	59,643	93.90
Length of stay (day)		0.00		0.00
1–5	3,548	17.55	27,248	42.90
6–10	6,382	31.57	19,936	31.39
>10	10,288	50.89	16,332	25.71
Surgery				
No	18,268	90.36	51,275	80.73
Yes	1,950	9.64	12,241	19.27
Residential type				
Rural	2,803	13.86	35,137	55.32
Urban	17,415	86.14	28,379	44.68

^*^N and percentages were based on study samples (unweighted). UEBMI, Urban employee basic medical insurance; URBMI, Urban resident basic medical insurance; TSP, Total self-paying; and CMI, Commercial medical insurance.

### Changes in Chinese middle-aged and older adults affected by single disease and by multimorbidity

**Figure 1:**
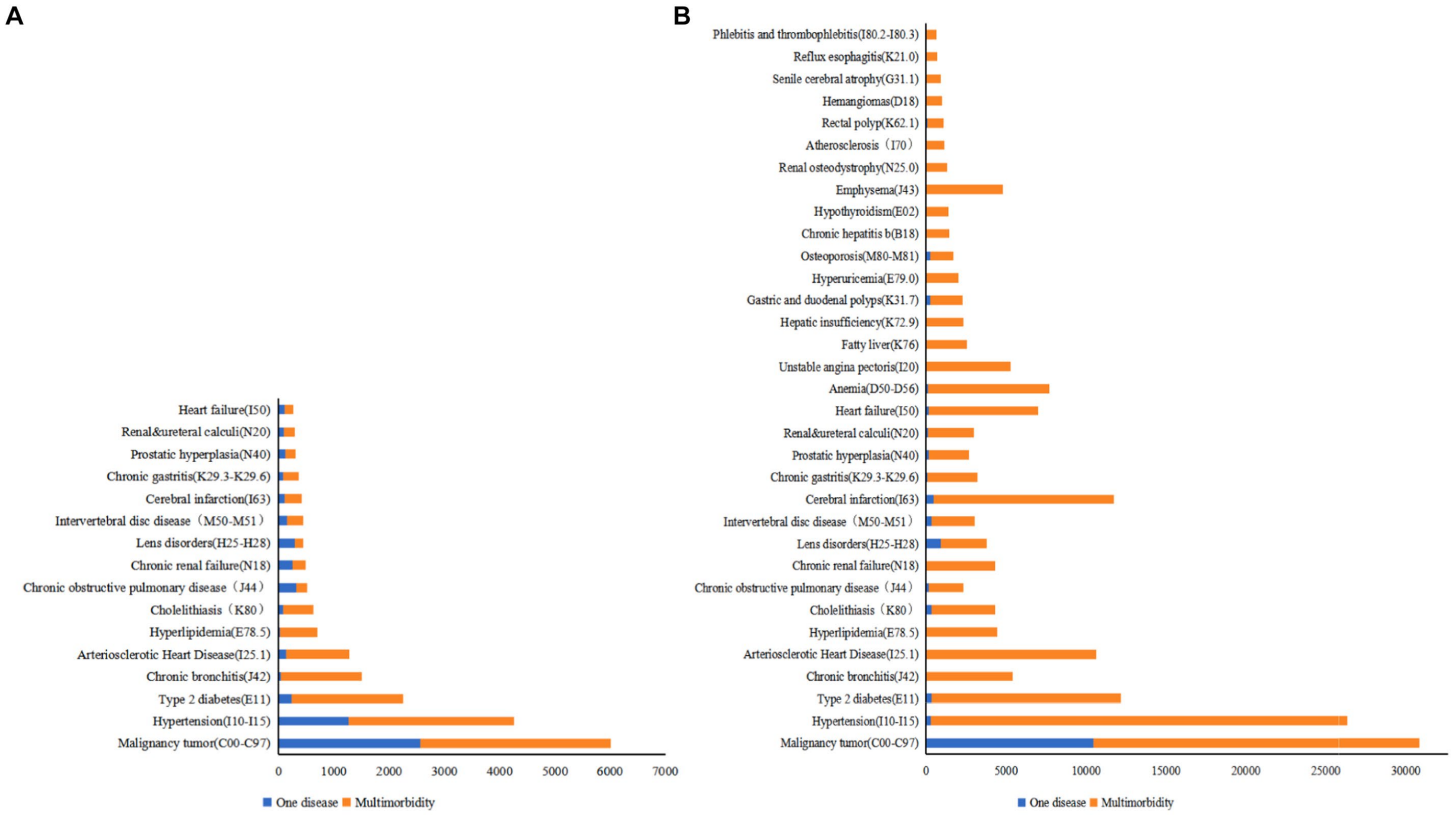
Changes in the number of Chinese middle-aged and older adults affected by single disease and by multimorbidity (*n*). **(A)** Chronic diseases among middle-aged and older people, 2013. **(B)** Chronic diseases among middle-aged and older people, 2023.

The study standardized chronic diseases based on clinical characteristics and ICD-10. After excluding chronic diseases with detection rates less than 1%, 16 chronic diseases were identified in 2013. Out of these, 12,820 (70.74%) were multimorbidity, with the median medical cost of 8,216.74 RMB (IQR: 5802.28–15,737). For a single disease, the median medical costs were 5,979.05 RMB (IQR: 4706.07–8890.01). In 2023, 33 chronic diseases were identified, 48,672 (76.63%) were multimorbidity. The median medical cost of multimorbidity were 7247.96 RMB (IQR: 5014.63–15434.06), while 5979.05 RMB (IQR: 4706.07–8890.01) in patients with single disease (Figure 1).

### Results of LCA

To explore the patterns of multimorbidity among chronic diseases, this study initially conducted LCA on data from 14,304 patients with various chronic conditions in 2013. The analysis covered 16 different chronic diseases and conditions, progressively fitting models from one to seven potential categories. By comparing each model’s BIC and G^2^, it was found that the model with six categories showed the best data fit (G^2^ = 2,953) and the lowest BIC (BIC = 140652.1). No significant improvements in model fitting were observed with additional categories, thus six categories were deemed most suitable for the 2013 dataset. A similar methodological framework was applied to the 2023 data, involving 48,673 patients and 33 chronic diseases/conditions. The models were fitted from 1 to 10 potential categories. Model effectiveness evaluation: the same statistical standards revealed that the model with nine latent categories, achieving a G^2^ of 67441.87 and minimizing BIC to 876923.3, offered the optimal balance between data fit and model complexity. Therefore, nine categories were selected as the ideal model for the 2023 data. To enhance the transparency and scientific integrity of these conclusions, Supplementary Tables S1, S2 detail the BIC and G^2^ indices for each model across the years, providing a rigorous statistical basis for category selection.

**Figure 2:**
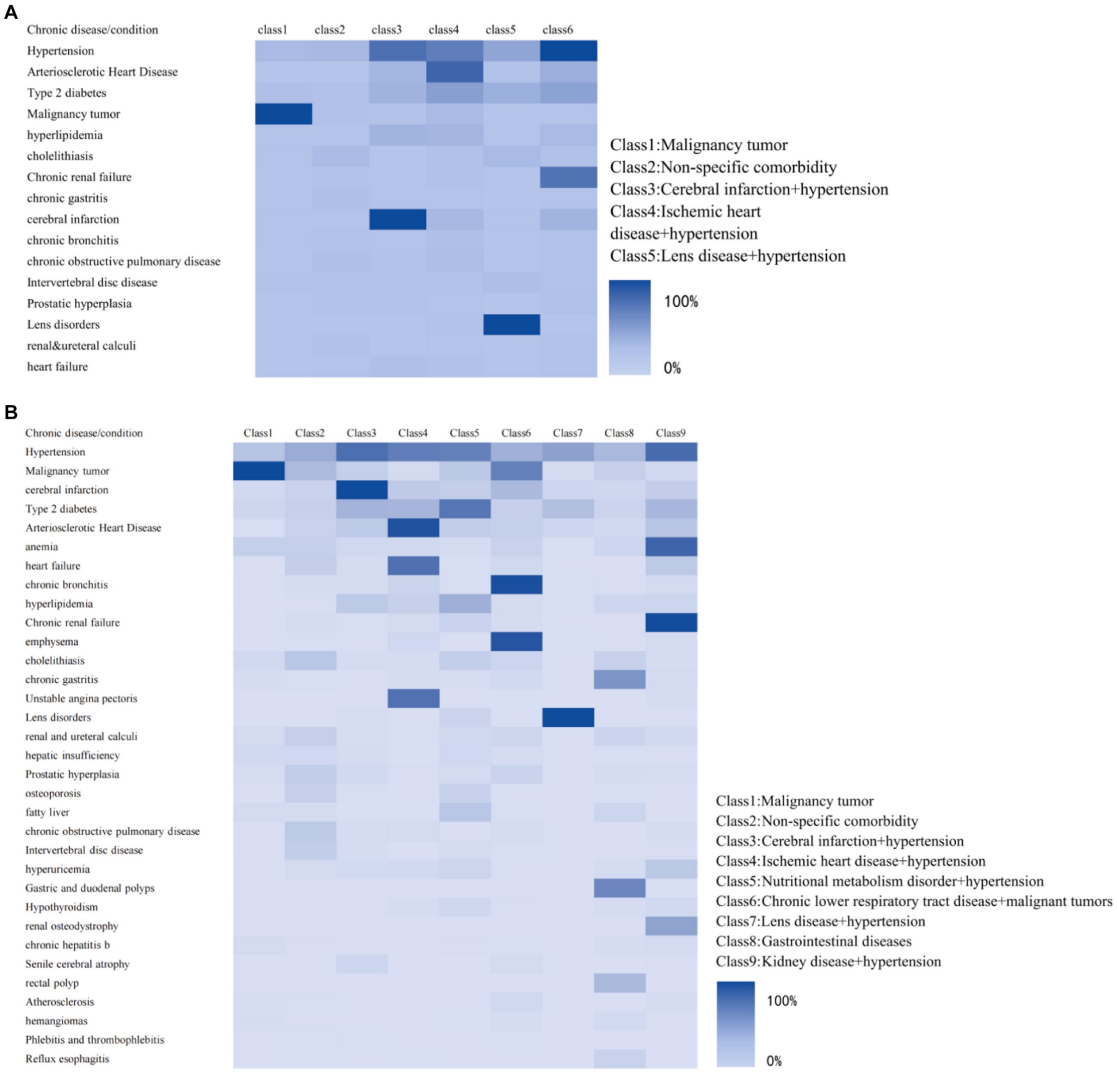
Latent category conditional probability heat maps for middle-aged and older patients. **(A)** Potential categories for 2013. **(B)** Potential categories for 2023.

The great likelihood method was used to estimate the posterior probability of each diagnosis being classified into a specific category (Supplementary Tables S3, S4). The magnitude of each posterior probability was then compared to determine the potential category to which the entry should belong. Each potential category was named based on the clinical features of the disease and the organs involved. For the sake of comparison, we assigned the same name to groups with similar probabilities of responding to chronic diseases in both fitted groups. Finally, the text describes different patterns of comorbidity, including malignant tumor pattern (mainly primary or secondary tumor combinations), cerebral infarction and hypertension pattern (including cerebral infarction, hypertension, and type 2 diabetes), ischemic heart disease and hypertension pattern (including arteriosclerotic heart disease and heart diabetes), kidney disease and hypertension pattern (including chronic renal failure, anemia, hypertension, type 2 diabetes, and renal osteodystrophy), and a non-specific comorbidity pattern (involving multiple chronic diseases with a low response probability). The patient presents with a nutritional metabolism disorder and hypertension pattern, specifically Type 2 diabetes, hyperlipidemia, and fatty liver. Additionally, they have a chronic lower respiratory tract disease and hypertension pattern, including chronic bronchitis, emphysema, and malignancy tumor. There is also a gastroenterology issue present with hypertension. Gastrointestinal diseases, including chronic gastritis, gastric and duodenal polyps, and rectal polyps, were observed in 2023. The latter three patterns were newly identified (Figure 2, Table 2).

**Table 2:** LCA categorization results and differences between 2013 and 2023.

Multimorbidity pattern	2013			2023		
	*N*	%	Medical cost	*N*	%	Medical cost
Malignancy tumor	6,636	32.82	7555.55 (5064.56, 12344.32)	25,120	39.55	7549.195 (4392.61–17379.5)
Non-specific comorbidity	6,453	31.92	8834.56 (5490.351, 15915.69)	7,134	11.23	7966.925 (4683.19–12854.59)
Ischemic heart disease + hypertension	4,308	21.31	8518.71 (5512.806, 14332.28)	6,620	10.42	8259.915 (5516.79–17840.80)
Cerebral infarction + hypertension	1,666	8.24	8274.02 (5380.695,13793.59)	7,084	11.15	8214.42 (5502.2–14,022)
Kidney disease + hypertension	653	3.23	7774.69 (5229.443, 12243.52)	3,072	4.84	6688.455 (4387.305–10563.02)
Lens disease + hypertension	502	2.48	10557.36 (8097.176, 18617.5)	3,223	5.07	5819.56 (5519.485,6205.78)
Nutritional metabolism disorder + hypertension				4,269	6.72	5540.15 (4310.3–7839.17)
Chronic lower respiratory tract disease + malignant tumors				3,921	6.17	7850.74 (4958.73–15127.74)
Gastrointestinal diseases				3,073	4.84	6195.78 (4803.17, 7789.22)

The LCA yields six potential categories in 2013 and nine potential categories in 2023, and the table is sorted according to the high percentage of potential categories in 2013, with medical costs expressed using median (IQR).

### Results of GRA

In 2013, diagnostic tests fees have the highest association with each hospitalization cost for different chronic comorbidity patterns (0.934), followed by comprehensive medical services (0.926) and drugs (0.848), while consumables have the lowest association (0.653). However, by 2023, fees for comprehensive medical services have the highest association with total hospitalization costs (0.927), fees for diagnostic tests drop to the second highest association (0.844), followed by other fees (0.82), and the smallest association with treatment costs (0.733) (Tables 3, 4).

**Table 3:** Results of GRA correlation analysis of medical costs for multimorbidity pattern among middle-aged and older people in 2013.

Multimorbidity pattern	CMS	DEF	TC	DC	COC	OC
Malignancy tumor	0.918	0.997	0.809	0.943	0.965	0.876
Cerebral infarction + hypertension	0.972	0.983	0.716	0.986	0.675	0.85
Ischemic heart disease + hypertension	0.867	0.812	0.71	0.645	0.334	0.611
Lens disease + hypertension	0.915	0.904	0.921	0.809	0.591	0.834
Kidney disease + hypertension	0.915	1	0.957	0.98	0.845	0.939
Non-specific comorbidity	0.968	0.909	0.918	0.724	0.508	0.681
*R*	0.926	0.934	0.838	0.848	0.653	0.799
Rank	2	1	4	3	6	5

The numbers in the table are relatedness (R), which denotes the intrinsic link between the total medical cost and the sub-costs in different co-morbidity patterns. 0 < *R* < 0.35 is considered as weak correlation, 0.35 ≤ *R* < 0.65 is considered as medium correlation, and 0.65 ≤ *R* < 1 is considered as strong correlation. CMS, Comprehensive medical services; DEF, Diagnostic examination fee; TC, Treatment cost; DC, Drug costs; COC, Cost of consumables; OC, Other costs; R, Relatedness.

**Table 4:** Results of GRA correlation analysis of medical costs for different chronic disease co-morbidity patterns among middle-aged and older people in 2013.

Multimorbidity pattern	CMS	DEF	TC	DC	COC	OC
Malignancy tumor	0.796	0.529	0.378	0.339	0.359	0.997
Cerebral infarction + hypertension	0.912	0.808	0.65	0.644	0.688	0.693
Ischemic heart disease + hypertension	0.968	0.748	0.629	0.632	0.627	0.675
Lens disease + hypertension	1	0.957	0.923	0.922	0.923	0.869
Kidney disease + hypertension	0.978	0.918	0.833	0.836	0.834	0.856
Non-specific comorbidity	0.884	0.798	0.363	0.398	0.377	0.487
Nutritional metabolism disorder + hypertension	0.96	0.927	0.846	0.822	0.843	0.669
Chronic lower respiratory tract disease + malignant tumors	0.924	0.863	0.671	0.798	0.749	0.939
Gastrointestinal diseases	0.992	0.965	0.965	0.922	0.936	0.918
*R*	0.927	0.844	0.733	0.736	0.739	0.82
Rank	1	2	6	5	4	3

The numbers in the table are relatedness (R), which denotes the intrinsic link between the total medical cost and the sub-costs in different co-morbidity patterns. 0 < *R* < 0.35 is considered as weak correlation, 0.35 ≤ *R* < 0.65 is considered as medium correlation, and 0.65 ≤ *R* < 1 is considered as strong correlation. CMS, Comprehensive medical services; DEF, Diagnostic examination fee; TC, Treatment cost; DC, Drug costs; COC, Cost of consumables; OC, Other costs; R, Relatedness.

### Univariate analysis of factors

The chi-square test showed statistically significant differences in the associated factors of medical cost in demographic characteristics sociological characteristics: age, sex, type of health insurance, spouse (yes or no), length of stay in hospital (1–5, 6–10, ≥10), surgery (yes or no), and type of residence (rural or urban), and multimorbidity patterns, significant difference was found for all variables (Table 5).

**Table 5:** Distribution and differences of variables of middle-aged and older adults.

Characteristics	2013 (*N*, %)					2023 (*N*, %)				
	Q1	Q2	Q3	Q4	P	Q1	Q2	Q3	Q4	*p*
Age (years)					<0.001					<0.001
45~	875 (32.23)	682 (25.12)	543 (20)	615 (22.65)		1,307 (28.71)	1,032 (22.67)	1,077 (23.66)	1,136 (24.96)	
50~	1,702 (27.37)	1,562 (25.12)	1,439 (23.14)	1,515 (24.36)		4,888 (27.47)	4,058 (22.81)	4,250 (23.89)	4,596 (25.83)	
60~	1,492 (22.86)	1,611 (24.68)	1,714 (26.26)	1,710 (26.2)		5,168 (24.57)	5,210 (24.77)	5,328 (25.33)	5,327 (25.33)	
70~	779 (21.23)	950 (25.89)	1,033 (28.15)	908 (24.74)		3,442 (22.02)	4,266 (27.3)	4,077 (26.09)	3,844 (24.6)	
≥80	206 (18.93)	248 (22.79)	328 (30.15)	306 (28.13)		1,074 (23.81)	1,313 (29.11)	1,147 (25.43)	976 (21.64)	
Sex					<0.001					<0.001
Men	2,206 (21.44)	2,373 (23.07)	2,601 (25.28)	3,107 (30.2)		7,952 (23.93)	7,892 (23.75)	8,520 (25.63)	8,872 (26.69)	
Women	2,848 (28.68)	2,680 (26.99)	2,456 (24.73)	1,947 (19.61)		7,927 (26.18)	7,987 (26.38)	7,359 (24.3)	7,007 (23.14)	
Health insurance type					<0.001					<0.001
UEBMI	3,490 (24.42)	3,786 (26.49)	3,684 (25.77)	3,334 (23.32)		6,765 (24.4)	7,785 (28.08)	6,890 (24.85)	6,284 (22.67)	
URBMI	1,261 (26.51)	1,026 (21.57)	1,081 (22.73)	1,388 (29.18)		8,272 (24.14)	7,852 (22.92)	8,760 (25.57)	9,379 (27.37)	
TSP	291 (27.42)	216 (20.36)	252 (23.75)	302 (28.46)		826 (57)	221 (15.25)	209 (14.42)	193 (13.32)	
CMI	12 (11.21)	25 (23.36)	40 (37.38)	30 (28.04)		16 (20)	21 (26.25)	20 (25)	23 (28.75)	
Spouse					<0.001					0.017
No	144 (28.45)	138 (27.27)	126 (24.9)	98 (19.37)		701 (18.1)	1,216 (31.4)	1,001 (25.85)	955 (24.66)	
Yes	4,910 (24.91)	4,915 (24.93)	4,931 (25.02)	4,956 (25.14)		15,178 (25.45)	14,663 (24.58)	14,878 (24.95)	14,924 (25.02)	
Length of stay (day)					<0.001					<0.001
1–5	2,267 (63.89)	821 (23.14)	338 (9.53)	122 (3.44)		11,103 (40.75)	8,938 (32.8)	5,016 (18.41)	2,191 (8.04)	
6–10	2,197 (34.42)	2,107 (33.01)	1,597 (25.02)	481 (7.54)		4,098 (20.56)	5,221 (26.19)	6,740 (33.81)	3,877 (19.45)	
>10	590 (5.73)	2,125 (20.66)	3,122 (30.35)	4,451 (43.26)		678 (4.15)	1,720 (10.53)	4,123 (25.24)	9,811 (60.07)	
Surgery					<0.001					<0.001
No	5,037 (27.57)	4,865 (26.63)	4,335 (23.73)	4,031 (22.07)		15,773 (30.76)	12,929 (25.22)	13,908 (27.12)	8,665 (16.9)	
Yes	17 (0.87)	188 (9.64)	722 (37.03)	1,023 (52.46)		106 (0.87)	2,950 (24.1)	1,971 (16.1)	7,214 (58.93)	
Residential type					<0.001					<0.001
Rural	809 (28.86)	671 (23.94)	618 (22.05)	705 (25.15)		8,844 (25.17)	9,484 (26.99)	8,740 (24.87)	8,069 (22.96)	
Urban	4,245 (24.37)	4,382 (25.16)	4,439 (25.49)	4,349 (24.97)		7,035 (24.79)	6,395 (22.53)	7,139 (25.16)	7,810 (27.52)	
Multimorbidity pattern					<0.001					<0.001
One disease	346.125 (21.30)	373.75 (23)	422.5 (26)	471 (29)		3,919 (26.43)	5,923 (39.94)	2,984 (20.12)	2,004 (13.51)	
Malignancy tumor	1,726 (26.01)	1,673 (25.22)	1,429 (21.54)	1,274 (19.19)		5,518 (28.67)	3,561 (18.5)	4,137 (21.49)	6,033 (31.34)	
Non-specific comorbidity	1,406 (21.85)	1,351 (20.99)	1,501 (23.33)	1,692 (26.3)		1,431 (26.18)	988 (18.07)	1,769 (32.35)	1,279 (23.4)	
Ischemic heart disease + hypertension	930 (21.60)	984 (22.91)	1,028 (23.89)	1,015 (23.57)		992 (19.55)	920 (18.13)	1,431 (28.22)	1,730 (34.11)	
Cerebral infarction + hypertension	378.88 (22.74)	397 (23.85)	379 (22.8)	376 (22.58)		972 (17.91)	1,225 (22.56)	1,797 (33.1)	1,434 (26.43)	
Kidney disease + hypertension	160 (24.50)	167 (25.63)	145 (22.25)	128 (19.58)		705 (29.95)	562 (23.89)	693 (29.46)	393 (16.7)	
Lens disease + hypertension	49 (9.89)	74 (14.84)	170 (33.89)	167 (33.21)		108 (4.37)	2,194 (88.83)	143 (5.77)	25 (1.02)	
Nutritional metabolism disorder + hypertension						1,149 (35.14)	1,125 (34.39)	687 (20.99)	310 (9.49)	
Chronic lower respiratory tract disease + malignant tumors						700 (23.31)	647 (21.53)	808 (26.88)	850 (28.28)	
Gastrointestinal diseases						592 (25.12)	947 (40.22)	703 (29.87)	113 (4.78)	

Using 2023 costs as a benchmark, we standardized 2013 costs using the Consumer Price Index (CPI). In addition, inpatient costs were categorized into four quartiles (0–25, 25–50, 50–75, and 75–100%), i.e., the cost range in 2013 was categorized as less than RMB 5364.12 (Q1), 5364.12–8,303.73 (Q2), 8303.73–14027.74 (Q3), and greater than 14027.74 (Q4), and the cost range for 2023 is categorized as below RMB4810.43 (Q1), 4810.43–7121.03 (Q2), 7121.03–13479.75 (Q3), and greater than 13479.75 (Q4).

### Sensitivity analyses

The associations between multimorbitiy patterns and medical cost after excluding age ≥ 80, self-pay and commercial insurance separately between 2013 and 2023 were similar to their associations in fully-adjusted models (Supplementary Figures S3, S4). After adjusting for age, sex, health insurance type at admission, spouse, length of stay, surgery, and residential type, the 2013 quantile regression results showed that malignancy tumor, cerebral infarction and hypertension, ischemic heart disease and hypertension, kidney disease and hypertension, and non-specific comorbidity at the 25, 50, 75, and 90th percentiles of healthcare expenditures were mostly non-significant, while Lens disease + hypertension had a significant positive effect on hospitalization costs at the 75 and 90th percentiles. The 2023 quartile regression results indicated that all patterns of multimorbidity, except for Lens disease and hypertension, had a significant positive effect on medical costs in almost all quartiles (Supplementary Tables S3, S4).

### Associations between chronic multimorbidity patterns and medical cost

**Figure 3:**
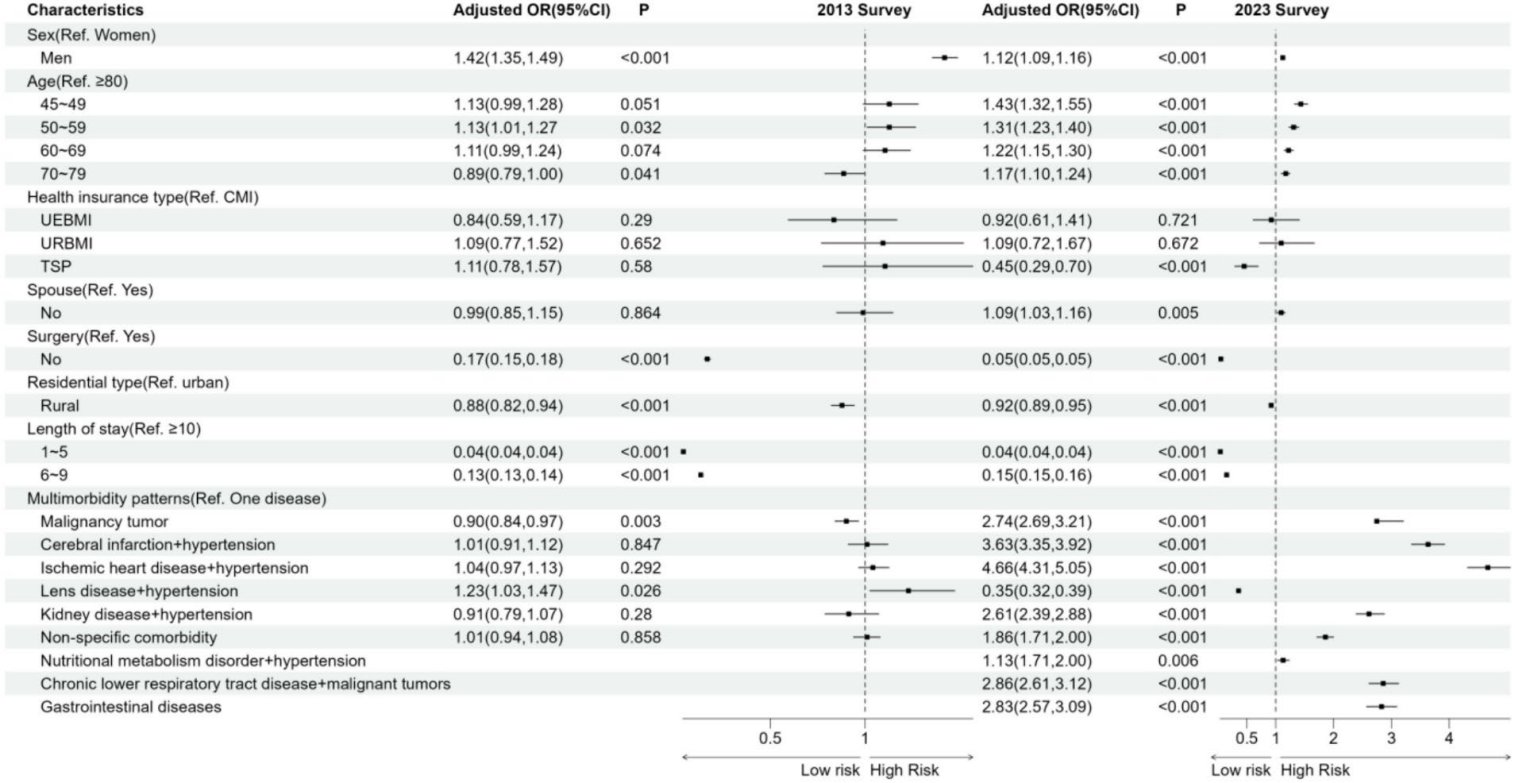
Associations between associations between chronic multimorbidity patterns and medical costin among Chinese middle-aged and older people. Results are from ordered multicategorical logistic regression, an odds ratio > 1 means that the variable is more likely to increase medical costs by one grade compared to the reference variable, and vice versa for an odds ratio < 1.

Figure 3 presents the results of the ordered logistic regression analysis. The study found that multimorbidity was significantly associated with increased direct healthcare expenditures in middle-aged and older adults with single diseases. In 2013, men, aged 50–59, lens disorders + hypertension had 1.42 (95% CI, 1.35, 1.49), 1.13 (95% CI, 1.01, 1.27), and 1.23 (95% CI, 1.03, 1.47) greater chances of increased medical costs, respectively. The study found that individuals who were between 70 and 79 years old (OR: 0.89, 95% CI: 0.79–1.00), did not undergo surgery (OR: 0.17, 95% CI: 0.15–0.18), resided in rural areas (OR: 0.88, 95% CI: 0.82–0.84), had a hospitalization period of 1–5 days (OR: 0.04, 95% CI: 0.04–0.04), 6–9 days (OR: 0.13, 95% CI: 0.13–0.14), or had malignant tumors (OR: 0.90, 95% CI: 0.84–0.94) were more likely to have lower hospitalization costs. Regression models from 2023 show that men (OR: 1.12, 95% CI: 1.09–1.16), ages 45–49 (OR: 1.43, 95% CI: 1.32–1.55), 50–59 (OR: 1.31, 95% CI: 1.23–1.40), 60–69 (OR: 1.22, 95% CI: 1.15–1.30), and 70–79 (OR: 1.17, 95% CI: 1.10–1.24), those without a spouse (OR: 1.09, 95% CI: 1.03–1.16), malignant tumors (OR: 2.74, 95% CI: 2.69–3.21), cerebral infarction + hypertension (OR: 3.63, 95% CI: 3.35–3.92), and ischemic heart disease + hypertension (OR: 4.66, 95% CI: 4.00–5.43) are all associated with increased risk. Patients with renal disorders and hypertensive disease had higher inpatient hospital costs (OR: 2.61, 95% CI: 2.39–2.88), as did those with non-specific disorders (OR: 1.86, 95% CI: 1.71–2.00), nutritional metabolism disorder + hypertension (OR: 1.31, 95% CI: 1.71–2.00), and gastrointestinal disorders (OR: 2.83, 95% CI: 2.57–3.09). Additionally, patients with Chronic lower respiratory tract disease + malignant tumors were found to have slightly higher inpatient hospital costs (OR: 1.09, 95% CI: 1.03–1.16). Patients who were total self-pay (OR: 0.45, 95% CI: 0.29, 0.70), did not undergo surgery (OR: 0.05, 95% CI: 0.05, 0.05), resided in rural areas (OR: 0.92, 95% CI: 0.89, 0.95), had hospitalization lasting 1–5 days (OR: 0.04, 95% CI: 0.04, 0.04), had hospitalization lasting 6–9 days (OR: 0.15, 95% CI: 0.15, 0.16), or had Lens disease + hypertension (OR: 0.35, 95% CI: 0.32, 0.39) were more likely to have lower hospitalization costs. The Hosmer–Lemeshow test showed a good fit for the regression model (*p* = 0.658, *p* = 1.171).

## Discussion

Our study shows that the detection rate of chronic disease multimorbidity and a new trend among middle-aged and older Chinese people increased from 2013 to 2023, with the pattern of multimorbidity based on LCA increasing from 6 to 9. Ordered logistic regression showed that multimorbidity was associated with higher healthcare expenditure compared with patients with a single disease. More importantly, the odds of incurring higher medical costs were not consistent across multimorbidity patterns, so we explored the heterogeneity of different multimorbidity patterns in the structure of medical costs using GRA. To the best of our knowledge, this is the first study to assess the prevalence and common patterns of multimorbidity among middle-aged and older Chinese at the complete clinical diagnosis level, and to explore changes in multimorbidity patterns and their associations with health care costs over a 10-year period. Our findings emphasize the need for a deeper understanding of the changing trends, treatment and prevention strategies, and a clearer analysis of the economic impact of multimorbidity, which can help the government to further develop strategies for the prevention and treatment of chronic diseases and to reform health insurance and health care systems.

The study found that the detection rate of multimorbidity increased from 70.74 to 76.63% over a period of 10 years, which was higher than in previous studies (19, 22). Possible differences in prevalence could be attributed to the fact that previous studies were based on predefined types of chronic diseases, which may have underestimated the actual prevalence of chronic diseases. Additionally, the use of hospital data in this study may have contributed to an increase in chronic disease diagnoses. Of the two cross-sectional studies that identified specific patterns of chronic disease multimorbidity, the top three were all Malignancy tumor pattern, Cerebral infarction + hypertension pattern and Ischemic heart disease + hypertension pattern. The latter two were among the most commonly identified multimorbidity patterns in previous studies (10, 23). This is similar to the results of other studies. The prevalence of cardiovascular disease in China is continuously rising. It is projected that there are currently 330 million CVD patients in China, including 13 million strokes and 11.39 million cases of coronary heart disease (24). Hypertension is a significant risk factor for ischemic heart disease and cerebral infarction. Prolonged high pressure damages the endothelium of blood vessels, leading to atherosclerosis and narrowing of blood vessels. This increases the risk of forming blood clots, making it difficult for blood to flow smoothly to the brain and increasing the risk of cerebral infarction. Hypertension is an independent risk factor for the development of coronary heart disease. Research data suggest that for every 10–12 mmHg increase in systolic blood pressure or 5–6 mmHg increase in diastolic blood pressure, the likelihood of developing coronary heart disease increases by 20–28% (25).

Previous studies have seldom reported pattern of malignant tumor. According to the International Agency for Research on Cancer of the World Health Organization, there will be over 35 million new cancer cases by 2050, which represents a 77% increase over the estimated 20 million cases in 2022, and aging and growth of the population are associated with a rapidly increasing global cancer burden (26, 27). A review in China found that daily expenditures for all common cancers increased 3–7 times from 1996 to 2014 (28). We believe that attention should be paid to the economic burden arising from maintenance and palliative care for malignant tumors.

The 2023 survey added three new multimorbidity patterns of chronic lower respiratory tract disease + malignant tumors, nutritional metabolism disorder + hypertension, and gastrointestinal diseases. The chronic lower respiratory tract disease + malignant tumors has rarely been reported before, probably because of the high prevalence of smoking and air pollution in China in recent years, which has led to the prevalence of chronic lower respiratory diseases (29–34). The pathogenesis of chronic lower respiratory disease and lung cancer, the world’s most common cancer, are related and may influence each other to some extent (35, 36). Our study discovered that hypertension and type 2 diabetes had high response probabilities in multimorbidity patterns and were significant components of multimorbidity patterns, such as cerebral infarction + hypertension, ischemic heart disease + hypertension, lens disorders + hypertension, renal disorders + hypertension, and nutritional metabolism disorder + hypertension. Similar trends have been observed in studies from multiple countries (37, 38), which revealed that hypertension and diabetes are the most common multimorbidity. It is hypothesized that these conditions make individuals more susceptible to accumulating other chronic diseases. Therefore, it is necessary to enhance screening and diagnosis of high-risk groups with risk factors and intervene in the early stages of chronic disease development to slow down the progression and reduce the incidence of complications.

The study also showed that the impact of chronic diseases on medical costs varies depending on the multimorbidity patterns. Additionally, the risk of cost was found to be higher in patients with ischemic heart disease + hypertension, as well as cerebral infarction +hypertension. This finding suggests that individuals with more diseases within these two multimorbidity patterns face higher medical costs. There are several reasons for this phenomenon. With the improvement of economic levels and medical technology, interventional treatments such as stent implantation and bypass grafting have become the preferred treatment modality, significantly increasing healthcare expenditures compared to conservative treatments. GRA indicates that in 2013, the total hospitalization costs for both modalities were primarily associated with drug and diagnostic test costs. However, in 2023, these costs changed to comprehensive medical service costs, while the correlation of consumable costs increased, confirming this trend. It is possible that the reversal of the trend in medical costs for lens disorders + hypertension in the two survey results is related to China’s centralized drug purchasing policy. This policy involves national government departments collecting the demand for medical supplies from public medical institutions at all levels across the country and negotiating with drug and consumable manufacturers through a group purchasing program. Centralized purchasing of medicines is a process in which the national government collects the demand for medicines and supplies from public medical institutions at all levels across the country. The government then negotiates with the manufacturers of medicines and supplies in a “group purchasing” manner to lower the prices of medicines and supplies, thereby reducing the burden of patients’ medical expenses. The hospitalization cost of the lens disease and hypertension model is highly correlated with the cost of consumables. Expensive high-end intraocular lenses (IOLs) have been included in the collective purchasing catalog, and the significant reduction in the cost of consumables has led to a reduction in the cost of cataract surgeries. Similar to the results of other studies, all other chronic disease models in the study have an elevated risk of medical expenditure. All other patterns of multimorbidity had a significant positive impact on medical cost in 2023 compared to patients with a single disease, similar to the findings of other studies (39–42). Patients with multimorbidity tend to require more treatments, tests, and medications, leading to higher healthcare costs (43–45).

At the demographic and sociological level, male inpatients, and no spouse had a positive effect on total hospitalization costs, which may be due to different physiological structures, lifestyles, and life stresses. Both men and women experience life and work pressure, but men may be more likely to engage in unhealthy behaviors such as smoking, drinking, and staying up late. These factors can contribute to more serious health issues for male patients, resulting in higher healthcare costs compared to women (46). Compared to patients with spouses, nulliparous patients have lower social support (47), and are more likely to experience mental health problems (48, 49), which may increase their risk of illness and hospitalization costs. However, further research is needed to explore the specific mechanisms involved. Age ≥ 80, total self-pay, no surgery, rural residence, and low inpatient days had lower risk of medical cost. A hospital survey in China found that treatment choices for older patients were associated with quality of life, modestly prolonged life expectancy, dying at home and lower health care costs, older patients with chronic multimorbidity often have complex conditions and poor tolerance, they may prefer low-cost conservative treatments to minimize pain and financial burden caused by medical treatment (50). Patients who are fully self-funded are not covered by health insurance due to various factors, including household registration, fixed contributions, lack of health awareness, and limited access to information. Additionally, some patients may prefer conservative treatment to avoid incurring large medical expenses (51, 52). The duration of hospitalization and the necessity of surgery are the primary factors that affect hospitalization costs. Therefore, hospitals should focus on improving medical technology while implementing effective measures to enhance efficiency and avoid unnecessary medical procedures. This will help to reduce the length of hospital stays and control the growth of hospitalization costs (53). Compared to patients residing in suburban areas, those living in urban areas have greater access to healthcare resources and higher ability to pay. This results in greater access to high-quality healthcare services, including accurate diagnosis, thorough physical examinations, and timely treatment. This may explain the higher healthcare expenditures for urban patients (24, 54).

The findings from our study on the evolving multimorbidity patterns and associated healthcare costs among middle-aged and older populations in China carry significant implications for healthcare policy. As the prevalence of multimorbidity increases with an aging population, it becomes imperative to focus on managing and preventing chronic diseases. Policymakers should optimize the allocation of medical resources to better address the rising number of chronic disease patients. Enhancing primary healthcare facilities’ capabilities to manage multiple chronic conditions could reduce the burden on tertiary hospitals and provide patients with more accessible, continuous care (55). Our study also reveals a trend where overall healthcare costs have decreased, yet expenses linked to specific multimorbidity patterns remain high. This observation should prompt policymakers to meticulously analyze the cost-effectiveness of different disease combinations and devise targeted medical payment policies. For high-cost disease combinations, the government could implement targeted funding support and incentives to control medical costs and enhance patient care quality (56). Furthermore, in light of ongoing reforms in China’s healthcare system, these findings encourage a review of current health insurance policies to ensure they effectively cover the needs of high-risk groups, especially the older with multiple diseases (57). Ensuring that insurance policies adequately meet the needs of these vulnerable groups is crucial for enhancing the efficiency and equity of the healthcare system.

Our study has several limitations. Firstly, our findings are derived from data collected at a tertiary public hospital, which serves over half of Xiangyang’s residents. Therefore, extrapolation of our results should be approached with caution. Secondly, due to the limited duration of the study, only diseases with a detection rate of ≥1% were analyzed, leaving the associations of less prevalent diseases unexplored. Additionally, all participants were from the same healthcare system, which limits the interpretation of our results due to the absence of a control group. Future studies should consider incorporating control groups to more accurately assess the impact of healthcare system changes. Moreover, the impact of the COVID-19 pandemic, coinciding with the study period, was not specifically analyzed. Our focus remains on long-term trends in multimorbidity and medical costs, rather than the immediate effects of global health crises. Future studies could employ prospective cohort research and include control groups to better understand how COVID-19 affects these multimorbidity patterns and healthcare costs. Finally, due to data constraints, potential risk factors such as BMI, smoking, alcohol consumption, social status, and annual income were not analyzed. Despite these limitations, the methodologies employed in this study could be valuable for future research.

## Conclusion

Multimorbidity patterns are common among middle-aged and older Chinese patients and have become increasingly complex in the last decade. Three new multimorbidity patterns have been identified: chronic lower respiratory tract disease + malignant tumors, nutritional metabolism disorder + hypertension, and gastrointestinal disease, as well as a less previously reported malignancy tumor pattern. Patients with multimorbidity were more likely to have higher medical costs. The highest risk was associated with cerebral infarction + hypertension and ischemic heart disease + hypertension. Identifying patterns of multimorbidity in chronic diseases and understanding their changing trends can aid in the development of new treatment guidelines and models of care to meet the healthcare needs of different subgroups. It is noteworthy that the average cost per hospitalization for multimorbidity patients has decreased over the past decade. Additionally, the correlation between diagnostic examination and drug costs and total healthcare expenditures has declined. Future research should examine the specific impacts of policy reforms on medical costs and service quality through comparative studies across various healthcare systems and regions. Such studies will enhance our understanding of effective medical resource management. Additionally, health reforms should prioritize the prevention, control, treatment, and management of multimorbidity through early intervention, health education, and robust health insurance policies, ultimately alleviating the economic burden on both individuals and society.

## Data availability statement

The datasets presented in this article are not readily available because the data that has been used are confidential. Requests to access the datasets should be directed to the corresponding author.

## Ethics statement

Ethical review and approval was not required for the study on human participants in accordance with the local legislation and institutional requirements. Written informed consent from the patients/participants or the patients’/participants’ legal guardian/next of kin was not required to participate in this study in accordance with the national legislation and the institutional requirements.

## Author contributions

CJ: Conceptualization, Data curation, Investigation, Methodology, Project administration, Resources, Validation, Visualization, Writing – original draft, Writing – review & editing. HL: Data curation, Writing – original draft. YoG: Data curation, Methodology, Writing – original draft. MG: Formal analysis, Investigation, Writing – review & editing. YiG: Data curation, Formal analysis, Writing – original draft. YuL: Writing – review & editing. RL: Data curation, Writing – original draft. MY: Writing – original draft. XiuL: Writing – original draft. YaL: Writing – review & editing. XiaL: Writing – review & editing. TH: Writing – review & editing. XiaoL: Writing – original draft. CH: Writing – review & editing. YX: Writing – review & editing. JL: Conceptualization, Data curation, Formal analysis, Investigation, Methodology, Supervision, Writing – original draft, Writing – review & editing.
