# Supplementary material for: Changes in patterns of multimorbidity and associated with medical costs among Chinese middle-aged and older adults from 2013 to 2023: an analysis of repeated cross-sectional surveys in Xiangyang, China
Source: Front Public Health. 2024 Aug 7;12:1403196. doi: 10.3389/fpubh.2024.1403196 (PMC11335498; doi:10.3389/fpubh.2024.1403196)
Supplement: Supplementary file 1 [file Data_Sheet_1.docx]

Supplementary File

**Figure S1.**Flowchart showing the selection of the subjects who were included in the final analysis,2013

**Figure S2.**Flowchart showing the selection of the subjects who were included in the final analysis,2023

**Table S1.** The fitting results of LCA model for NCDS in middle-aged and elderly people in 2013

**Table S2.** The fitting results of LCA model for NCDS in middle-aged and elderly people in 2023

**Figure S3.** Sensitiviy analysis of associations between multimorbitiy patterns and medical cost after excluding age≥80 ,self-pay and commercial insurance separately in 2013

**Figure S4.** Sensitiviy analysis of associations between multimorbitiy patterns and medical cost after excluding age≥80 ,self-pay and commercial insurance separately in 2023

**Table S3.** Results of quantile regression analysis in 2013

**Table S4.** Results of quantile regression analysis in 2023


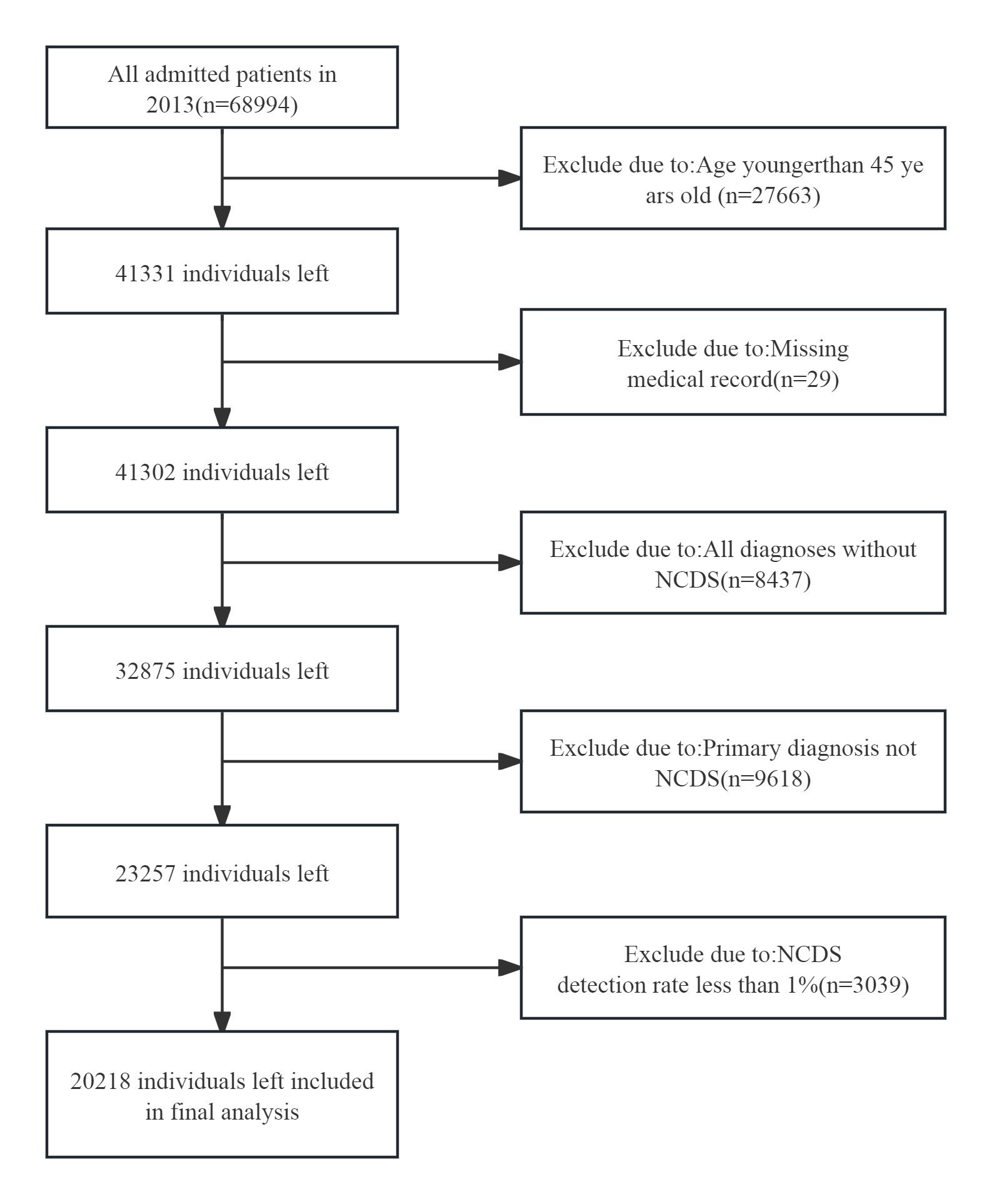


Figure S1.Flowchart showing the selection of the subjects who were included in the final analysis,2013


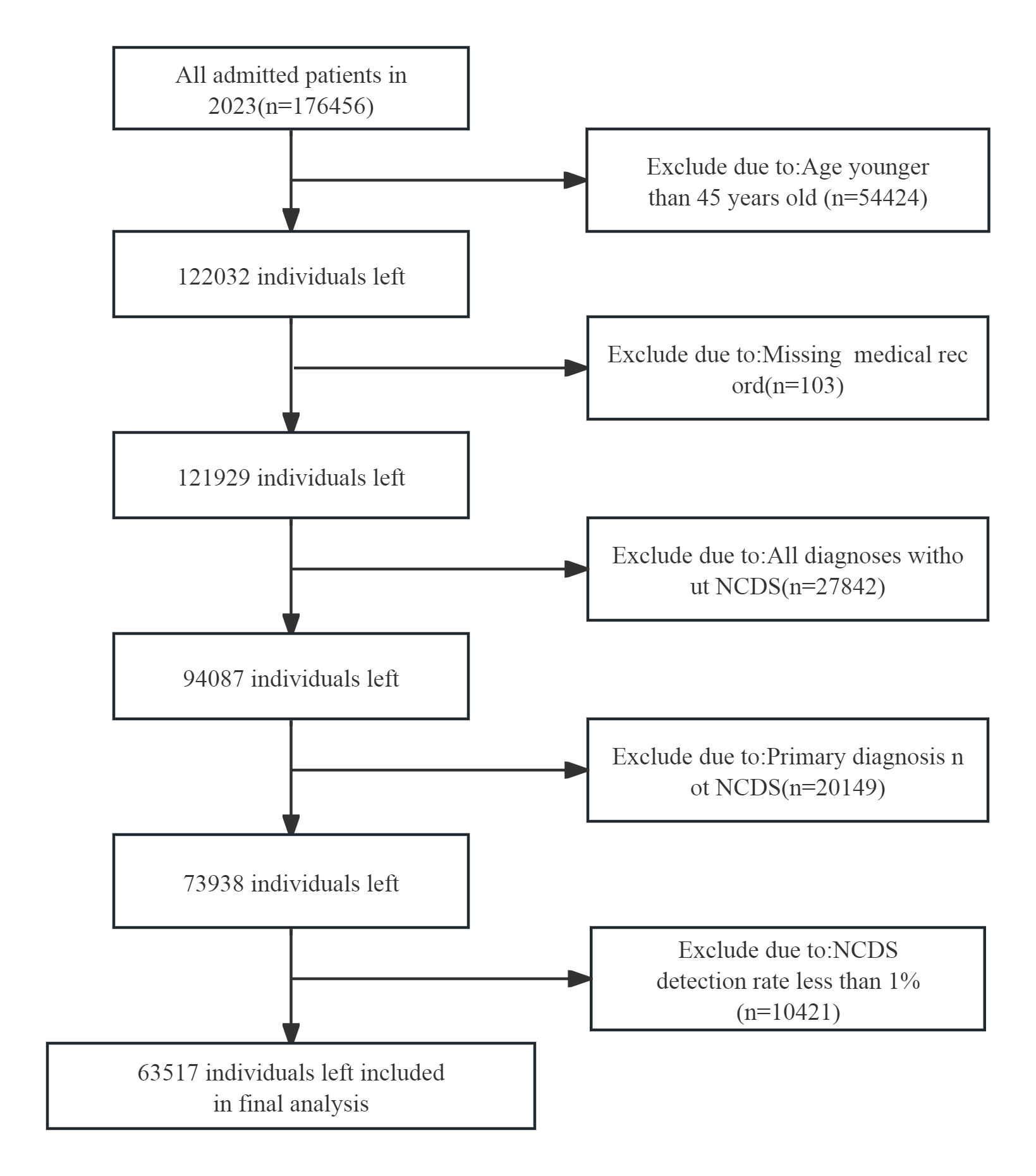


Figure S2.Flowchart showing the selection of the subjects who were included in the final analysis,2023

Table S1. The fitting results of LCA model for NCDS in middle-aged and elderly people in 2013

| Class | Log_likelihood | G^2^ | AIC | BIC |
| --- | --- | --- | --- | --- |
| 1 | -75579.52 | 14699.60 | 151199.0 | 151357.3 |
| 2 | -71177.51 | 5895.56 | 142437.0 | 142761.5 |
| 3 | -70283.57 | 4107.72 | 140691.1 | 141181.8 |
| 4 | -69988.71 | 3517.97 | 140143.4 | 140800.3 |
| 5 | -69860.64 | 3261.75 | 139929.2 | 140752.3 |
| 6 | -69706.42 | 2953.40 | 139662.8 | 140652.1 |
| 7 | -69598.48 | 2837.51 | 139489.0 | 140744.4 |

Table S2. The fitting results of LCA model for NCDS in middle-aged and elderly people in 2023

| Class | Log_likelihood | G^2^ | AIC | BIC |
| --- | --- | --- | --- | --- |
| 1 | -504887.3 | 204163.8 | 1009851 | 1010195 |
| 2 | -474739.6 | 143868.3 | 949633.1 | 950330.7 |
| 3 | -460273.2 | 114935.6 | 920778.4 | 921829.3 |
| 4 | -451297.1 | 96983.36 | 902904.2 | 904308.3 |
| 5 | -446303 | 86995.27 | 892994.1 | 894751.5 |
| 6 | -445260.3 | 84909.8 | 890986.6 | 893097.4 |
| 7 | -438416 | 71221.2 | 877376 | 879840.1 |
| 8 | -437529.5 | 69448.2 | 875681 | 878498.4 |
| 9 | -436526.3 | 67941.87 | 873752.7 | 876923.3 |
| 10 | -435149.3 | 66473.87 | 871076.4 | 879426.5 |

Figure S3. Sensitiviy analysis of associations between multimorbitiy patterns and medical cost after excluding age≥80 ,self-pay and commercial insurance separately in 2013


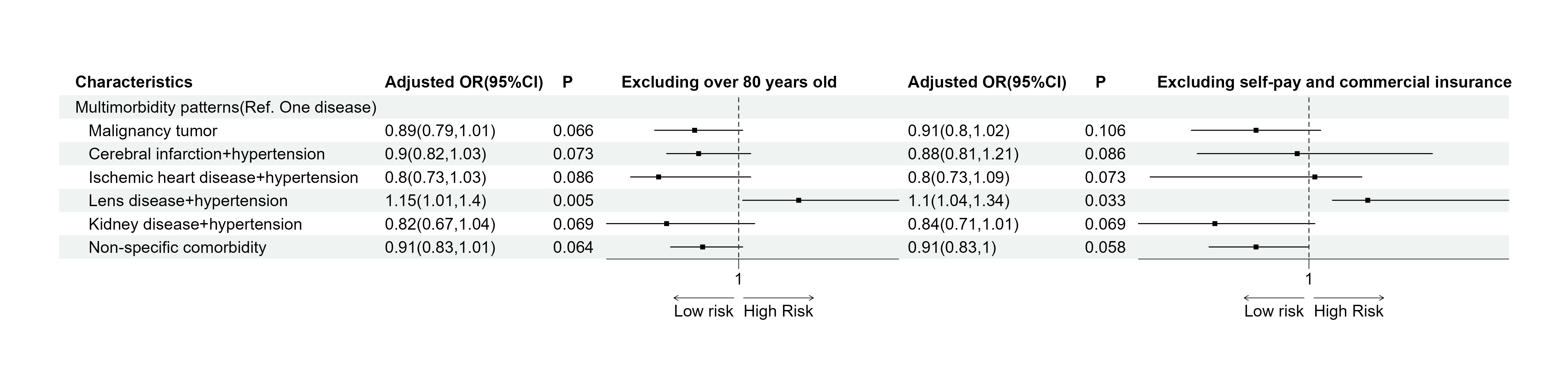


Ordered multicategorical logistic regression,adjusted for age, sex,type of health insurance, spouse (yes or no), length of stay in hospital (1-5, 6-10, ≥10), surgery (yes or no), and type of residence (rural or urban).

Figure S4. Sensitiviy analysis of associations between multimorbitiy patterns and medical cost after excluding age≥80 ,self-pay and commercial insurance separately in 2023


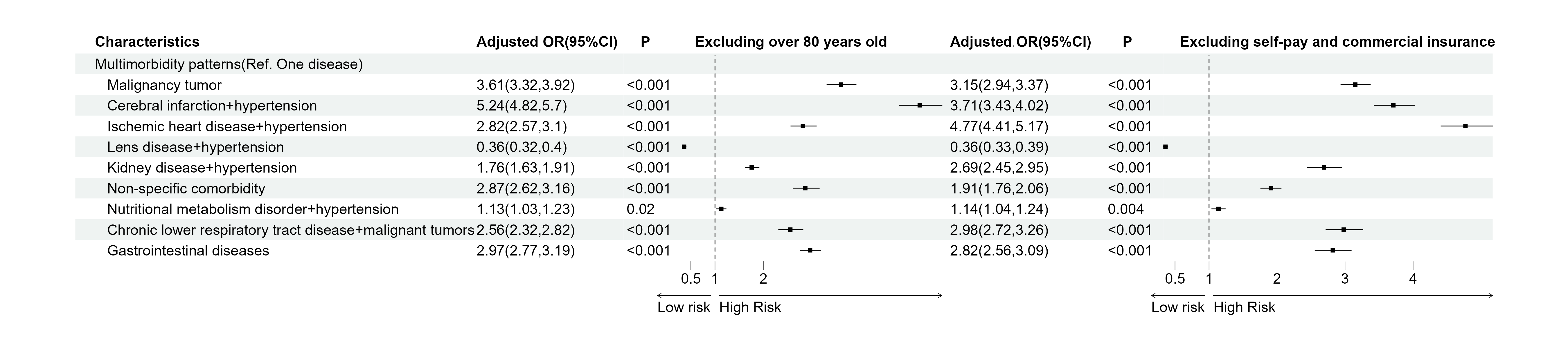


Ordered multicategorical logistic regression,adjusted for age, sex,type of health insurance, spouse (yes or no), length of stay in hospital (1-5, 6-10, ≥10), surgery (yes or no), and type of residence (rural or urban).

Table S3. Results of quantile regression analysis in 2013

| Multimorbidity Pattern | 25 percentile | | | 50 percentile | | | 75 percentile | | | 90 percentile | | |
| --- | --- | --- | --- | --- | --- | --- | --- | --- | --- | --- | --- | --- |
|  | Coeff | 95%CI | *P* | Coeff | 95%CI | *P* | Coeff | 95%CI | *P* | Coeff | 95%CI | *P* |
| Malignancy tumor | 30 | -175,235 | 0.776 | -286 | -644,72 | 0.000 | -693 | -1399,14 | 0.055 | -1328 | -2956,300 | 0.110 |
| Non-specific comorbidity | -111 | -290,67 | 0.221 | -353 | -625,81 | 0.117 | -457 | -1062,147 | 0.138 | -478 | -1936,979 | 0.520 |
| Ischemic heart disease+hypertension | -344 | -506,-182 | <0.001 | -452 | -805,499 | 0.011 | -999 | -1549,-449 | <0.001 | -1220 | -2535,95 | 0.069 |
| Cerebral infarction+hypertension | -198 | -416,19 | 0.074 | -696 | -1526,134 | 0.000 | -255 | -1507,997 | 0.690 | 1130 | -2085,4344 | 0.491 |
| Kidney disease+hypertension | -281 | -577,15 | 0.063 | -468 | -826,-111 | 0.100 | -361 | -1376,655 | 0.486 | -1282 | -3110,546 | 0.169 |
| Lens disease+hypertension | -107 | -275,60 | 0.209 | -335 | -608,-63 | 0.078 | 437 | 398,542 | 0.002 | 428 | 401,562 | 0.008 |

quantile regression,adjusted for age,sex, type of health insurance, spouse (yes or no), length of stay in hospital (1-5, 6-10, ≥10), surgery (yes or no), and type of residence (rural or urban).

Table S4. Results of quantile regression analysis in 2023

| Multimorbidity Pattern | 25 percentile | | | 50 percentile | | | 75 percentile | | | 90 percentile | | |
| --- | --- | --- | --- | --- | --- | --- | --- | --- | --- | --- | --- | --- |
|  | Coeff | 95%CI | p | Coeff | 95%CI | p | Coeff | 95%CI | p | Coeff | 95%CI | p |
| Malignancy tumor | 3068 | 2721, 3415 | <0.001 | 2752 | 2555,2950 | <0.001 | 3960 | 3636,4283 | <0.001 | 6122 | 5552,6692 | <0.001 |
| Non-specific comorbidity | 2348 | 2002,2693 | <0.001 | 958 | 756,1160 | <0.001 | 560 | 216,903 | 0.0014 | 258 | -317,832 | 0.37968 |
| Cerebral infarction+hypertension | 3132 | 2775,3489 | <0.001 | 2657 | 2427,2886 | <0.001 | 2560 | 2220,2901 | <0.001 | 3471 | 2653,4289 | <0.001 |
| Ischemic heart disease+hypertension | 3660 | 3289,4032 | <0.001 | 3613 | 3385,3841 | <0.001 | 3274 | 2965,3583 | <0.001 | 2463 | 1925,3001 | <0.001 |
| Nutritional metabolism disorder+hypertension | 1937 | 1592,2282 | <0.001 | 72 | -128,271 | 0.483 | -276 | -638,87 | 0.13599 | -170 | -803,462 | 0.59827 |
| Chronic lower respiratory tract disease  +malignant tumors | 3076 | 2708,3445 | <0.001 | 2531 | 2270,2792 | <0.001 | 3533 | 3011,4056 | <0.001 | 4682 | 3627,5737 | <0.001 |
| Lens disease+hypertension | -1607 | -1967,-1247 | <0.001 | -5489 | -5775,-5204 | <0.001 | -11889 | -12465,-11314 | <0.001 | -17585 | -18765,-16406 | <0.001 |
| Gastrointestinal diseases | 3400 | 3043,3757 | <0.001 | 2450 | 2237,2662 | <0.001 | 2308 | 1971,2645 | <0.001 | 1883 | 1351,2415 | <0.001 |
| Kidney disease+hypertension | 2905 | 2544,3267 | <0.001 | 2214 | 1986,2443 | <0.001 | 1647 | 1327,1968 | <0.001 | 1262 | 665,1860 | <0.001 |

quantile regression,adjusted for age, sex,type of health insurance, spouse (yes or no), length of stay in hospital (1-5, 6-10, ≥10), surgery (yes or no), and type of residence (rural or urban).
